# Centering and Shifting of Centrosomes in Cells

**DOI:** 10.3390/cells9061351

**Published:** 2020-05-29

**Authors:** Anton V. Burakov, Elena S. Nadezhdina

**Affiliations:** 1 A. N. Belozersky Institute of Physico-Chemical Biology, M. V. Lomonosov Moscow State University, 119991 Moscow, Russia; antburakov@belozersky.msu.ru; 2 Institute of Protein Research of Russian Academy of Science, Pushchino, 142290 Moscow Region, Russia

**Keywords:** microtubule, actin, actomyosin, dynein, micropatterned substrate, basal-apical, planar polarity, aster, cilia, tissue in situ

## Abstract

Centrosomes have a nonrandom localization in the cells: either they occupy the centroid of the zone free of the actomyosin cortex or they are shifted to the edge of the cell, where their presence is justified from a functional point of view, for example, to organize additional microtubules or primary cilia. This review discusses centrosome placement options in cultured and in situ cells. It has been proven that the central arrangement of centrosomes is due mainly to the pulling microtubules forces developed by dynein located on the cell cortex and intracellular vesicles. The pushing forces from dynamic microtubules and actomyosin also contribute, although the molecular mechanisms of their action have not yet been elucidated. Centrosomal displacement is caused by external cues, depending on signaling, and is drawn through the redistribution of dynein, the asymmetrization of microtubules through the capture of their plus ends, and the redistribution of actomyosin, which, in turn, is associated with basal-apical cell polarization.

## 1. Introduction

The centrosome got its name from the fact that it often occupies a central position in the cell; it was discovered even in the early works of cytologists who studied blastomeres or leukocytes using the iron hematoxylin staining method by Heidenhein [1]. Radial microtubules diverging from the centrosome create a beautiful aster, and the idea arose that such microtubule arrangements zoning the cytoplasm indicate the paths to and from the center of the cell. It seems obvious that a clear radial arrangement of microtubules can facilitate the transport of various cell components to the local central region, where the centrosome itself is located, often followed by the Golgi surrounding it. Oddly enough, this assumption was not verified experimentally. Recently, it was shown [2] that the randomization of microtubules leads to the inhibition of the formation of the primary cilia at the centrosome and to a delayed restoration of the Golgi after its dispersion, which can be associated with inefficient intracellular transport that got lost on randomized microtubules. The problem likely requires further study. Distracted from admiring the picture of microtubules aster (hereafter aster), we can ask ourselves a few questions about the location of the centrosome in the cell.

(1) Is the centrosome always centered in the cell?

(2) The nucleus is also located approximately in the center of cells. How are the centrosome arrangement and the nucleus arrangement related?

(3) What forces put the centrosome in the center of the cell and hold it there—or, on the other hand, push it out of the center? In other words, what are the molecular mechanisms of centrosome centering and its displacement throughout the cell?

(4) Finally, why do the cells need the exact positioning of the centrosome?

In recent years, significant progress has been made in finding answers to these questions. Here, we will try to analyze and summarize the available information.

## 2. Localization of Centrosomes in the Cells

### 2.1. Centrosomes in Substrate-Spread Cells

**Figure 1:**
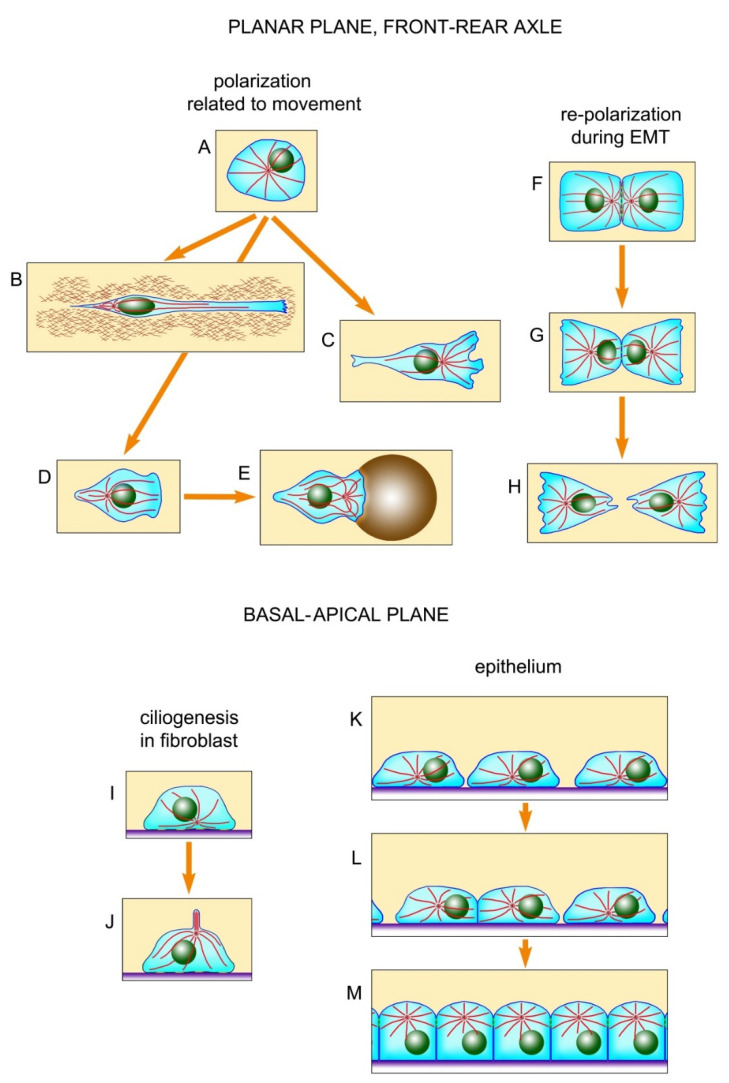
Different variants of centrosome displacement from a central position in the cell. (**A**)—a central position of the centrosome in symmetric non-polarized cell. (**B**)—fibroblast move surrounded by a non-adhesive substrate. (**C**)—fibroblast move freely. (**D**)—lymphocyte movement. (**E**)—formation of an immune synapse by lymphocyte. (**F**–**H**)—stages of epithelial-mesenchymal transition. (**I**,**J**)—formation of primary cilium. (**K**–**M**)—centrosome shifting to the apical part of the cells during epithelial differentiation.

A centrally located centrosome is intuitively expected to be found in actively proliferating cultured cells that are spread upon the substrate, or in the spread leukocytes. Such cells usually appear to be two-dimensional, and the location of the centrosome in them also appears in two-dimensional space. The location of the centrosome along the vertical axis of the cell (closer to or farther from the substrate) attracts significantly less attention; however, as we will see later, it is no less significant. A thorough analysis of the location of the centrosome in mouse embryonic fibroblasts was carried out in [3]. The authors analyzed cells of arbitrary shape growing in a sparse monolayer, cells in a confluent monolayer, and cells with a predefined form on a micropatterned substrate: disk-shaped or triangular (an isosceles triangle with an aspect ratio of 7:4). The position of the centrosome in the cells with respect to the centroid was studied. A centroid is a point inside a geometric figure, whose coordinates correspond to the arithmetic average of the coordinates of all points in the figure. In other words, it is defined as the center of mass. It turned out that in the cells in the confluent monolayer, as well as in the round-shaped cells molded by micropattern, the centrosome was located closer to the centroid (Figure 1A) than it was in triangular cells or different-shaped cells in a sparse culture. The centrosome was disposed from the centroid by less than 10% of the average cell radius in about 75% of round-shaped cells and in only 40% of triangular cells or those that freely move on the substrate. The authors also noted that in triangular cells, the centrosome shifted to the base of the triangle (the wide part of the cell), which, in their opinion, imitates the displacement of the centrosome to the leading edge of the cells moving along the substrate. Such a movement is characteristic of fibroblasts or astrocytes and was noted in several studies of centrosome positioning [4,5].

It is believed that the displacement of the centrosome to the leading edge of the cell, where it is located in front of the nucleus, organizes the antero-posterior axis of the cell through the axis of the nucleus-centrosome and sets the direction of locomotion (Figure 1C). Such an arrangement of the centrosome is usually shown in schematic drawings of a moving fibroblast. Meanwhile, in some types of cells, or when cells move along certain substrates, the centrosome may not even move forward, and may even localize in the back part of the cell behind the nucleus [4]. In particular, when fibroblasts move along thin strips of fibronectin substrate surrounded by a non-adhesive surface, that is, in elongated cells moving in one dimension, the centrosome is localized in their posterior part [6,7,8] (Figure 1B). The centrosome is similarly located in the lateral line primordium cells of zebrafish embryo in situ, in the posterior part of the cells in relation to the direction of movement [6]. These apparent exceptions, which did not fit into the classical centering scheme earlier, were recently explained in the article by Jimenez et al. [9]: these exceptions are apparently due to the asymmetry of the actin cytoskeleton, i.e., the external contour of the cells should not be taken into account; rather, the internal contour of the submembrane actin cytoskeleton should be considered (see below). In addition to the centrosome, the nucleus in the cells tends to be located centrally, which undoubtedly affects the location of the centrosome. On the one hand, the nucleus and centrosome are attracted to each other (reviewed in [10]); on the other hand, the nucleus can push the centrosome to the periphery of the cell. This problem can be solved by studying the location of the centrosome in cells lacking a nucleus—i.e., cytoplasts.

The most accurate analysis of the centrosome location in cytoplasts was carried out in a recent paper by Jimenez et al. [9]. The authors studied the positioning of the centrosome in the cytoplasts obtained by the classical method (centrifugation in the presence of cytochalasin B), where the nucleus could not distort the location of the centrosome; which, in addition, were spread on the micropatterned substrate, where the attached and expanded cytoplasts had a predetermined substrate shape. The adhesive sites of the substrate had the same area. It turned out that, in cytoplasts of different shapes, the centrosome always chooses a location close to the centroid. In this case, the centrosome can be remoted from other centers of the geometric figure—for example, the center of a circle inscribed in a triangle. Interestingly, the displacement of the centrosome from the centroid toward the base of the triangle was significant only in the triangle with an aspect ratio of 7:4; in a more elongated triangle (9:2 ratio), the centrosome remained in the centroid. In L-shaped or sickle-shaped cells, the centrosome shifted from the centroid to the side of the concave edge. Thus, we can conclude that the centrosome can recognize—or determine—the shape of the cells and that the centrosome is located in the centroid mainly in round or, on the other hand, strongly elongated cells, while in cells of another shape it can be noticeably displaced from the centroid. It was previously suggested that the centriole can determine the shape of cells [11], but this was related to such a specific object as *Chlamydomonas*, whose shape is far from being as variable as the shape of fibroblasts. Rather, the centrosome specifically recognizes the shape, i.e., cell edges, and, as we will see later, it considers the actin cortex.

### 2.2. Centrosomes in Cells In Situ and in Organ Cultures

The location of centrosomes in cells in situ or in organ cultures has not been systematically studied, although in situ centrosomes have been observed in many objects, in different animals, and in humans. Centrosomes were studied mainly in the single-layer epithelium or epithelial-like layers of cells: the intestinal epithelium [12,13,14,15,16], ependyma, kidney [17,18], vascular endothelium [19], Corti organ [20], and organ cultures of the breast ducts and renal ducts, *Drosophila* embryonic tissues, mouse embryo during gastrulation, and cells of fish embryos and *Xenopus* [21,22,23,24]. A separate layer of observations relates to the centrosome in neurons and glia (see below). Unfortunately, the location of centrosomes in liver cells (except on hepatocyte cultures [25]) and in fibroblasts of the connective tissue matrix of various organs has not been practically studied. In single-layered cubic and cylindrical epithelia, the basal-apical and orthogonal to it (planar) planes of the cell projection can be distinguished. As a rule, researchers pay attention to the centrosome location relative to one of these planes. Based on published works, it can be concluded that the centrosome is usually localized in the apical part of epithelial cells [13,14,15,16,22,23], i.e., on the side of the cells facing the organ cavity and distant from the intercellular matrix (Figure 1K–M). That is, in the basal-apical projection, the centrosome is usually displaced from the center of the cell. It is interesting that in poorly differentiated epithelial cells, for example, in the intestinal crypt, the centrosome is located more precisely in the center of the cells, and only during differentiation, for example, in the intestinal villi, moves to the apical part [12,13,14,15,16]. The centrosome located in the apical part of the cells often organizes a basal-apical microtubule bundle [26], which provides transcytosis, i.e., the transfer of cargo from the apical to the basal surface of the cell and in the opposite direction. In differentiated cells, the centrosome sometimes loses the function of organizing microtubules, passing it to the non-centrosomal structures [12,13,14,15,16,26]. Moreover, it remains itself in the apical part of the cell, although few direct observations of this have been published. Sometimes, centrioles in differentiated cells degrade, and most of the cells in the intestinal villi do not have centrioles at all [14,15,16].

In many tissues, the centrosome forms the primary cilium protruding above the surface of the epithelial or endothelial layer or into the nephron duct [18]. In intestine cells cilia form only at embryos [16]. Upon the induction of cilia formation in cultured cells, during serum starvation, their centrosome also shifts to the part of the cell remote from the substrate [27], corresponding to the apical side of the epithelium (Figure 1I,J). In actively proliferating cultured cells, the centrosome is usually located in the part of the cell close to the substrate.

Special mention should be made of the planar cell polarity (PCP), which refers to the uniform polarization of cells within the plane of a cell sheet [28,29,30,31]. With this phenomenon, when it comes to projection onto a plane orthogonal to the basal-apical, centrosomes are often shifted to one edge of the cells. A pronounced PCP is observed, for example, in *Drosophila* wing cells during the formation of actin-supported protrusions—hairs, which, as well as centrosomes, are shifted to the distal edge of the cells. Therefore, the PCP phenomenon has been studied mainly in *Drosophila*, although in recent years a lot of work has been done on other objects. The formation of rows of stereocilia and uniformly oriented motile cilia also belong to the PCP phenomenon reviewed in [28,29,30,31]. We will not focus on this issue further. PCPs are more often found in the epithelium, although in many cases, it is not clear whether PCP exists in any epithelium, or whether the centrosome is shifted in a planar plane simply by chance. It is noteworthy that in the vascular endothelium, centrosomes are usually shifted to the edge of the cells facing the heart [20]. The shift of the centrosome to the leading edge of the fibroblasts is apparently also a special case of PCP due to the polarization of their actomyosin system, although the fibroblasts do not form an integral layer. During the epithelial-mesenchymal transition (EMT), which accompanies carcinogenesis and a series of events during embryogenesis, the centrosome in the epithelial cell moves from the apical to the basal part, which becomes anterior during further migration through the extracellular matrix (ECM), which is characteristic of mesenchymal cells [23] (Figure 1F–H). As mentioned previously, the location of the centrosome in fibroblasts and fibroblast-like cells has been studied mainly in culture; there are very few works that observed it in fibroblasts in situ [6].

Another particular case of natural displacement of the centrosome is the formation of an immune synapse (Figure 1D,E). It was noted that in polymorphonuclear leukocytes attached to a ferritin substrate, the centrosome is located mainly apically, and on the ferritin substrate with anti-ferritin antibodies, it moves to the basal part [32]. Centrosome displacement into the caudal region of the lymphocyte by which it attaches to the antigen-presenting cell or the target cell [33,34,35,36,37] is accompanied by the destruction of the connection of the centrosome to the nucleus and the formation of an immune synapse. The centrosome, coupled with the Golgi apparatus, always adjoins the immune synapse, organizing there an amplified bundle of microtubules. Similarly, in fibroblast-like cells that are polarized on the substrate, the centrosome is also located close to the Golgi, and both these organelles are often—although not always—shifted to the leading edge [5]. However, the centrosomal displacement to the immune synapse is usually compared to the shift of the centrosome to the apical edge of the cell during cilia formation [38] and not with the movement of the fibroblast.

Centrosome localization in mature neurons in situ is poorly understood. In differentiating neurons, the centrosome is often, but not always, located at the site of axon exit. During migration of the neuron, it appears at the leading edge, ahead of the nucleus. According to some reports, when a neuron moves, the centrosome first moves forward to the leading neurite; then, a nucleus is pulled behind it, surrounded by a network of microtubules. However, other studies have shown the independent movement of centrosomes and nuclei during the migration of neurons, when the centrosome is either in front of or behind the nucleus. Herewith, the centrosome moves uniformly, keeping up with the center of the cell, and the nucleus can make saltatory movements, either ahead of the centrosome or lagging behind it [39]. It should be noted that, in any case, the centrosome usually does not move far into the neuron’s outgrowth but remains in the cell body. Similar oscillatory movements are performed by the nucleus and centrosome when glioma cells move along thin lines coated with fibronectin [40]. Interphase centrosomes were mainly studied in in situ objects, though there are also many observations of the location of mitotic spindles in developing multilayer and single layer epithelium, where it is important for whether the cell divides vertically or horizontally relative to the epithelial layer, as well as along or across the axis of the tubular epithelial structures [17,41]. In the collecting duct in a developing kidney, cells divide along it [17]. The location of the spindles across the epithelial layer is important for the formation of the stratified epithelium. The spindles find the right position, navigating by the shape of the cells and, of course, by the properties of the cell cortex [42,43]. In particular, the location of the interphase centrosome and the presence of the primary cilia are important [17].

Thus, three types of centrosome positioning can be highlighted: (1) the centrosome is located close to the centroid of the projection of the cell onto the plane, which is especially characteristic of non-motile and non-polarized cultured cells, taking into account the thickness of the actin cortex; (2) a shift to one of the cell edges during cell polarization (PCP, EMT, etc.), as well as the formation of an immune synapse, neuritis growth, etc.; and (3) movement into the apical part of the cell, often associated with the growth of the primary cilia or other types of cilia; it is possible that the formation of an immune synapse also applies to this phenomenon [38]. All three types of centrosome positioning and movements may be due to different molecular mechanisms, although, of course, they are interconnected. It has been suggested, however, that both centering and centrosomal displacement can be determined by the same mechanism, depending on the absence or presence of at least small external cues [44].

### 2.3. Why Is the Determination of the Centrosome Position in the Cell Required?

As mentioned previously, the function of the central location of the centrosome in the cells is not clear. It is difficult to determine this function, if only because there are no experimental approaches for centrosome decentering that do not involve serious effects on the cytoskeleton. Therefore, we must confine ourselves to speculative discussions about the creation of a certain cell geometry, in particular, about the centrosome-nucleus axis in moving cells. It is possible that both the reason and purpose of the central location of the centrosome is to create a relatively symmetric radial microtubules network, which allows for the organizing of directional transport to the centrosome of the components necessary for its functioning, including cilia growth [2], i.e., provide “selfish” functions of the centrosome. The central location of mitotic spindles, in which the poles are formed by centrosomes, is more understandable. Mitotic spindles can be shifted using a micromanipulator or other similar actions (for example, Rappoport’s experiments [45] used the introduction of oil droplets) to the periphery of cells, in particular, large cells such as zygotes and blastomeres. The spindle shift leads to a shift of the cleavage furrow and the formation of daughter cells of unequal size. Numerous examples of asymmetric mitoses are well-known when the mitotic spindle is shifted from the center of cells due to some physiological mechanisms and, as a result of mitosis, unequal cells are formed: where the spindle pole was closer to the plasmalemma, the daughter cell turns out to be smaller. This phenomenon can be observed, in particular, during the division of *Drosophila* neuroblasts or mouse cerebellar cells [46]. Limits to the sizes of mitotic spindles and the regulation of their location are discussed in detail in several works [42,47,48]. The regulation of cell sizes is discussed in the review [49]; we will not dwell further on these topics.

The consequences of a shift of the centrosome from the center of the cell in the interphase are rather the opposite of the consequences of its shift in mitosis. If, in mitosis, a smaller pole of the fission spindle moves toward the plasmalemma, then in the interphase, the increased number of microtubules usually reaches the edge of the cell to which the centrosome moves, which can be clearly seen in the example of an immune synapse or even moving fibroblasts. The centrosome is often accompanied by the Golgi, at whose membranes additional microtubules are formed, which also go to the edge of the cell [50]. These microtubules appear to be used for enhanced transport to the outside of the vesicles containing, for example, metalloproteases that remodel ECM when the fibroblast moves [51], or enzymes that destroy the target cell of T-killer [37]. However, it has been shown that microtubules that are not associated with a centrosome are needed to polarize the endothelial cells of a growing vessel, while the centrosome, on the other hand, can inhibit cell polarization [52], especially in the presence of an excess of centrioles [53]. Another reason for the shift of the centrosome from the center to the edge of the cell is the formation of a primary or motile cilium or flagellum. It is believed that the same mechanisms are involved in the formation of the immune synapse and primary cilia, and they are discussed below. Finally, the centrosome shifts to the edge of the cell during PCP, for example, when strictly oriented hairs form on the wing cells of the *Drosophila*. The direct mechanisms of this shift remain unknown, although it has been established that, upstream, they depend on the dynamics of the actin cytoskeleton and on Wnt signaling. To shift the centrosome to the edge of the cell, it is necessary to initially shift it to the apical part, which always depends on actin [54].

In summary, the central arrangement of the centrosome arises due to the intrinsic properties of the cytoskeleton; we can assume that it is necessary for the selfish functions of the centrosome. The shift of the centrosome to the edge of the cell is regulated through the signal transduction pathways [5] and serves to fulfill the functions of the cells or the organ in which the cell is located.

## 3. Molecular Mechanisms That Determine the Location of Centrosomes in Cells

### 3.1. Central Location of the Centrosomes

**Figure 2:**
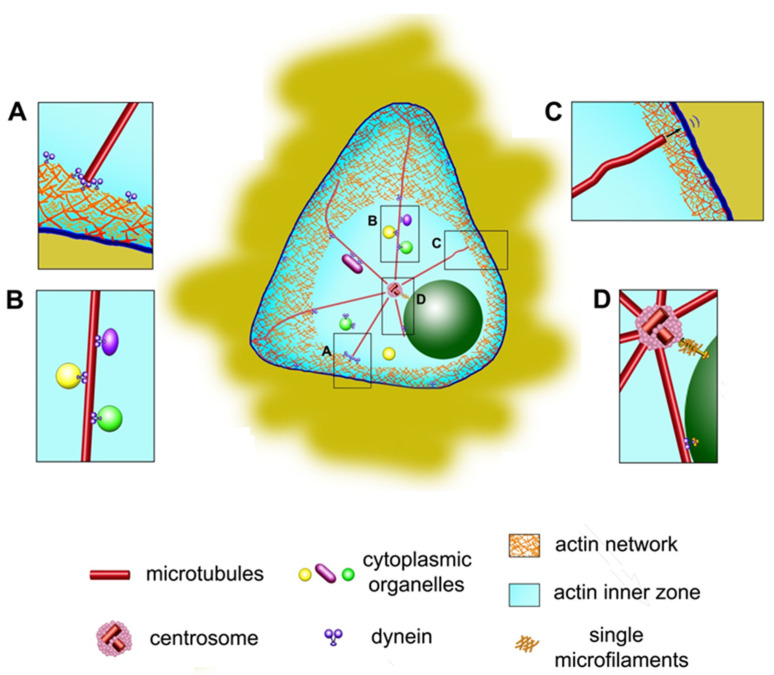
Geometry and mechanism of the centrosome centering. (**A**)—multiple dynein molecules pulling the microtubule from the cell periphery. (**B**)—pulling forces applied by dynein molecules anchored at the surface of cytoplasmic organelles along the microtubule. (**C**)—pushing forces generated by growing microtubule plus-end. The forces applied to microtubules by the actin cortical flow are not shown on this figure (**D**)—links between the centrosome and the nucleus. Central panel: note that the pulling forces are applied in actin inner zone only, and curved microtubules outside it do not contribute to centering [9].

Let us first consider why the centrosome is often located in the centroid in the projection of cells onto a plane. All researchers agree that the microtubule aster surrounding the centrosome plays the main role in centering. In particular, aster holds the centrosomes in the cells when enucleation is made under cytochalasin treatment [55]. Recent discoveries have shown that the centrosome initiates the polymerization of not only microtubules but also actin filaments [56]. However, most models of centrosome centering are based on the participation of cytoplasmic dynein interacting with microtubules. The role of dynein in centrosome centering has been shown in many experimental works [57,58,59,60], in which dynein activity was inhibited by low molecular weight inhibitors or the introduction of inhibitory proteins into the cell (for example, the CC1 fragment of the p150Glued protein) and a sharp centrosome displacement from the center was observed. It has been confirmed that the cell cortex in cultured cells contains many dynein molecules, coupled with its cofactor dynactin [61] and, in addition, many dynein molecules are located on intracellular membrane structures (Figure 2A,B). If *Dictyostelium* has two centrosomes in the binuclear cell, they form two independent asters, each occupying its own territory in the cytoplasm. Laser ablation of one of the asters leads to the rapid centering of the second aster, which is well-explained by the action of pulling forces from the side of the dynein [62]. Based on the general properties of the cytoskeleton, we can assume the effect of various forces on the aster: pushing forces from microtubule polymerization, pulling and pushing forces from microtubule motors, pushing and pulling forces from actomyosin interacting with microtubules, and pushing and pulling forces from actomyosin itself.

It was previously shown, with our participation, that the centrosome is kept at the center by a pulling force generated by dynein acting along microtubules and actin flow produced by myosin contraction [58]. These results were obtained from experiments with cells in which local microtubule destruction (LMD) was performed by the local application of nocodazole; moreover, the inhibition of the activity of dynein, myosin (actin flow inhibition), or microtubule dynamics were implemented. The results [58,63] are summarized in Table 1.

In the article of Zhu et al. [63], three possible mechanisms of centrosome centering were considered: (1) MTs can be pulled away from the centrosome by dynein molecules in the cortex, with the force being proportional to MT lengths; (2) MTs can be dragged toward the center of the cell by the actomyosin-driven inward flow of the actin network; and (3) MT growing plus-ends can bump into obstacles and push back on the centrosome. The total equation can be expressed as:

*F*_tot_ = *F*_dyn_ + *F*_act_ + *F*_push_, (1)

where *F*_dyn_, *F*_act_, and *F*_push_ are the total forces from dynein, actin-flow drag, and MTs’ pushing, respectively. Obviously, F_push_ depends on the f_push_, developed by a single microtubule, on the number of dynamic microtubules and the depth of dynamic instability; F_act_ depends on f_act_ (the effect of actin flow per unit length of microtubules) and the length of microtubules to the second degree; F_dyn_ depends on f_dyn_ (the strength of one dynein molecule) and the length of microtubules. The corresponding equations can be found in the Supplemental Material of [63]. To simplify, if the centrosome is shifted from the center of the cell, for example, to the right, then it has longer microtubules on the left, and then, both the outward (pulling to the left) F_dyn_ and inward (pushing to the right) F_act_ are larger on the left; shorter microtubules on the right develop increased F_push_, pushing to the left. The solution of the derived equations with parameters satisfying the experimental data was carried out. In the best way they corresponded per one microtubule to f_dyn_ proportional to 3 f_push_, and f_act_ proportional to 8 f_push_. In a computer simulation using the example of a round-shaped cell, the centrosome behavior described in Table 1 was fully reproduced. The centering mechanism was predicted to be robust: all that is needed for the centrosome centering is for the total dynein force to be greater than a modest threshold of 1 motor pulling per microtubule. The centrosome centering according to the described mechanism was reproduced on round, elliptical, square, and fan-shaped cell models. An interesting observation was made, reproduced both in vivo and in silico: when inhibiting dynein, the centrosome shifts to the long edge of the elliptical cell. This is a consequence of the strict dependence of the F_act_ value on the distance to the cell edge.

Questions that required further clarification were: whether the astral microtubules should terminate strictly at the plasmalemma or can slip along it, what is the role of other microtubule motor proteins that can develop pushing forces, can/should the microtubules be twisted near the centrosome, can/should they bend under the action of a pushing force, and can the dynein really develop such significant forces. Unfortunately, subsequent works paid little attention to the role of the actomyosin system in the behavior of centrosomes, although, as we see, this role is significant. It turned out to be convenient to study further issues by modeling both in silico and in vitro the microfabricated cells where isolated centrosomes and microtubule proteins were placed.

In the work of Wu et al. [60], the authors drew attention to the bending of microtubules after their severing with laser ablation and showed that this bending is determined by tensile and buckling dynein forces applied along the entire length of the microtubule. In silico modeling demonstrated that, in a small cell, the centrosome can occupy the center due only to the dynamics of microtubules (pushing forces), though in larger cells and a simulated viscous medium such as the cytoplasm, the pulling by dynein must apply [60]. In an in vitro system, centrosomes and tubulin were placed in microfabricated cells or inside liposomes, where an aster of microtubules formed, and the walls of the cells or liposomes were coated with dynein from the inside [64,65]. It was shown that dynein activates the catastrophes of microtubules growing to the dynein-covered barrier covered by it, and it significantly improves the centering of aster in a small volume. The asymmetry in the distribution of dynein leads to the decentering of such an aster. A particular problem is the centrosome centering in very large cells—for example, the movement of spermal aster associated with the male pronucleus and formed by the centrosome of the spermatozoon toward the female pronucleus located in the center of the oocyte of sea urchin, *Xenopus*, or *C. elegans* [66]. The microtubules of spermal aster may not reach the oocyte cortex with their ends, but the aster, along with the pronucleus, moves to the center of the oocyte at a fairly high speed [67,68,69,70]. This movement depends on microtubules and dynein, but does not depend on actomyosin [69,70]. It is believed that aster movement may be due to the dynein-dependent movement of small vesicles along microtubules to the center of the aster; vesicles move inward, and reactive forces pull microtubules outward [67,68,71,72]. Mutations and knockdown of genes encoding several proteins involved precisely in the minus-end transport (light chain of dynein DYRB-1, *rilp-1*, *rab-7*, and *rab-5*) in *C. elegans* led to the absence of centrosome centering in the zygote [71,72]. It should be noted that the spermal aster moves in the cytoplasm of the zygote along with the male pronucleus, i.e., the whole structure has a significant mass. Under this condition, the pushing forces from the microtubules are insufficient, and calculations show that the centration process cannot go without the action of dynein, though a dozen molecules of dynein are enough to develop the necessary forces [73]. Howard and Garzon-Coral [74], however, believe that pushing forces play the primary role in spindle centering, if microtubule buckling occurs at the plasmalemma. In the process of conjunction and the subsequent centering of pronuclei in the *C. elegans* zygote, the direction of movement of the male pronucleus changes, which is still waiting for an explanation. Simple phenomena, such as the accumulation of yolk at the vegetative pole of an egg, can lead to a change in the action vectors of various forces to the spindle of division and displace the spindle—and, after it, the division plane of the blastomeres [75]. Par1b/MARK kinase plays an important role in spindle positioning in the cell, regulating the interaction of microtubule ends with the cortex and balancing the pulling forces developed by dynein [25,76].

In Letort et al. [44], simulated in silico centrosome centering in cells of various shapes was performed without considering actomyosin forces, but taking into account the possibility of microtubules pivoting around the centrosome and gliding along the cell cortex. In all cases, the simulated microtubules were longer than the radius of the cell. It turned out that the centrosome-organized aster moves to the center of the round-shaped cell only by dynein pulling forces, with both a cortically located dynein and a dynein distributed over the cytoplasm. Using cell models of an ellipsoidal, rectangular, and triangular shape, it was found that with a uniform distribution of dynein in the cytoplasm, in all cases it is possible to achieve close to the central position of the centrosome. However, the greatest distance from the center of the aster to the cell centroid was obtained in triangles, especially in a triangle with a wide base. With the cortical arrangement of dynein in triangles, the simulated center of the aster was always shifted from the centroid. It also turned out that centering due to pushing forces was possible only in the absence of microtubule pivoting and gliding, i.e., with rigid fixation of both ends of the microtubules. In general, the aster centering was facilitated by a decrease in the stiffness of microtubules, an increase in their dynamics, and a diminution in the number. The introduction of asymmetric pulling forces produced by dynein molecules located on one of the sides of the round-shaped cell led to a sharp decentration of the aster [64,65].

### 3.2. Special Attention to the Role of Actin and Nuclei in Centrosome Positioning

Actin can play a special role in the location of centrosomes in a cell. Centrosome separation before mitosis depends not only from the intactness of the MT network, but also on the intactness of the actin filaments [77]. It was shown that centrosome separation in early prophase of mitosis (before NEB) in *Drosophila* embryos depends on the dynamics of actin filaments controlled by Arp2/3 and formin, although it does not depend on myosin [78]. Actin develops forces opposed to the kinesin Eg5, which promotes centrosome separation [79].

Jimenez et al. [9], also mentioned in the text above, showed that in a highly asymmetric cytoplast, there is a slight centrosome shift from the centroid, proportional to the asymmetry of the actin cytoskeleton. To describe the architecture of the actin cytoskeleton, the authors introduced new concepts of Actin Inner Zone (AIZ), i.e., a central zone of a cytoplast free of actin bundles, and AIZ geometric center, Actin Inner Center (AIC) (Figure 2, central panel). In a symmetric cell, AIC is the same as a centroid. It turned out that the position of the centrosome corresponds to the position of the AIC in both symmetric and asymmetric cells. If you treat a cell with a ROCK kinase inhibitor (Y27632), which suppresses the assembly of F-actin and retrograde actin flow, and removes actin bundles from the edges bordering AIZ, then actin is redistributed throughout the cell homogeneously, while AIC shifts and begins to coincide with the centroid—and the centrosome moves there too. Because the centrosome centering is due to the forces applied to the astral microtubules, the authors studied their architecture depending on the localization of the actin and found that microtubules are straight and radial in the AIZ (which indicates their tension and the force applied to them) but entangled outside this zone. Thus, even if MTs are knotty outside the AIZ, their radiality in the central zone will ensure successful centering. Experiments with cytoplasts of different sizes, but with approximately the same AIZ, showed that the accuracy of centrosome positioning depends on the size of the AIZ, and not on the whole size of the cell. When the microtubules are disassembled and the pulling forces applied by boundary positioned dynein/dynactin disappear, the centrosome leaves the AIC point, but not the AIZ zone, and randomly moves in it. Thus, the role of the actomyosin system is to determine the size and shape of the AIZ, which is the “legitimate field of activity” of the radial microtubules that pull the centrosome due to dynein forces and, thus, place it in the center of the AIZ. Microtubules extending from the centrosome can interact with actin through either specific crosslinkers or non-specific steric interactions [80]. In particular, dense-growing actin networks can apply pushing forces to microtubules [81,82].

One cannot help but mention another unobvious contribution of actin to centrosome positioning. In the work of Inoue et al. [83], it was demonstrated that actin filaments nucleating on the centrosome or in the immediate vicinity of it can affect the nucleation and growth of centrosomal microtubules. They physically block the formation of centrosomal microtubules at the earliest stages of their polymerization. The centrosomal actin affects the magnitude of the microtubule pushing forces generated by newly formed microtubules. Thus, not only does peripheral actin restrict AIZ involved in centrosome centering, but also centrosomal actin, which limits the nucleation of microtubules on the centrosome and, thus, affects the magnitude of the forces applied to it.

As already mentioned, the nucleus also seeks to occupy a central position in the cell, and to some extent competes with the centrosome for this position. The centrosome is associated with the nucleus (reviewed in [10]) and is usually located at a distance of about 0.2 microns from the nuclear envelope (Figure 2D). When the nucleus has a multilobulated morphology, as in neutrophils, the centrosome is located inside the nucleus courtyard [1,32,84]. The mutual arrangement of the nucleus and centrosome was investigated using above-described model of round-shaped and triangular-shaped single cells grown on a micropatterned substrate [3]. The association of the centrosome with the nucleus weakens, and the distance between these organelles increases with the disruption of microtubules by the action of nocodazole. When actin filaments were destroyed by the action of latrunculin B, the nucleus was displaced from the centroid and the distance from it to the centrosome increased as well. In round-shaped cells with depleted lamin A of the nuclear membrane, the distance between the centrosome and the nucleus increased significantly, probably due to the inability of the cytoskeletal structures to attach to the nucleus. In triangular cells, the distance between the centrosome and the nucleus increased especially noticeably under the action of LiCl, which inhibits the GSK-3β protein kinase, which, in turn, regulates the activity of dynein. Notably, in fibroblasts gliding along the substrate, the retrograde movement of the nucleus depends mainly on the dorsal actin filaments attached to the nucleus via the nesprin and SUN protein complexes of the nuclear membrane [85]. It is supposed that both the nucleus and the centrosome independently try to occupy a central position in the cell, with the position of the centrosome being determined mainly by the microtubule network, and the position of the nucleus by the actin network, but the nucleus and centrosome are connected by a separate bundle of microtubules [3]. However, we believe that they are also linked by actin and that the location in the cell of both the nucleus and the centrosome is determined by the coordinated action of cytoskeletal structures, which was demonstrated in [40]. Mainly, the movement of a nucleus through a cell depends on both actomyosin and microtubules-dynein, depending on the direction of movement [86].

The positioning of the centrosome in the cell also depends on some of its proteins, in particular, TBCCD1, from the family of tubulin chaperones. The TBCCD1 knockout results in the centrosome losing its clear positioning in the cells and its increased distance from the nucleus, although the centrosome’s ability to organize microtubules does not change [87]. The distance from the centrosome to the nucleus also increases when hypoxia and inflammation act on the cells, which leads to their release of ATP and the activation of the A2 purinergic receptor. Signaling from A2b goes through Epac1/RapGef3 and Rap1B [88].

### 3.3. Mechanisms of Off-Center Centrosome Positioning in Cells

**Figure 3:**
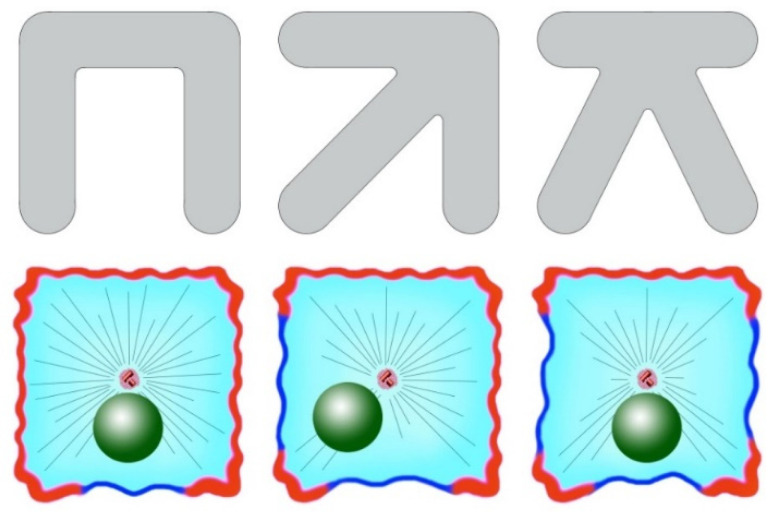
Actin polarization affects the location of the nucleus. The cells were seeded in adhesive patterns of different forms (top row). The nucleus-centrosome axis in all cases was directed toward more adhesions (shown with red), with the centrosome always located in the centroid of the cell and with the nucleus shifted toward free edges (shown with blue). Based on data described in [89].

As mentioned previously, to disrupt the central positioning of the centrosomes in cells, cytoskeleton rearrangements are necessary: asymmetrization of the actomyosin system and/or the arrangement of dynein or its activating factors. Supporting data were also obtained with cells growing on a micropatterned substrate [89]. RPE cells were seeded to crossbow-shaped adhesive patterns, which forced them to take on the shape of a fan, with the sides being unattached to the substrate. This led to an asymmetric distribution of actin, actin-binding proteins, and microtubule plus ends. The nucleus in these cells was somewhat shifted from the centroid to the narrow part of the cell, and the axis of the nucleus-centrosome formed in a manner directed from the narrow to the wide part of the fan. The authors also examined cells seeded in adhesive patterns in the form of letters X, Π, and K, and the form of short arrows. In all cases, the cells externally had a square shape, but were attached to the substrate, and their non-attached edges were located differently (Figure 3). It turned out that the nucleus-centrosome axis in all cases was directed toward more adhesions, with the centrosome always located in the centroid of the cell and with the nucleus shifted toward free edges. The authors believe that proteins that bind actin and microtubules, such as APC, concentrate on the adhesive edges of the cells and capture the plus ends of the microtubules, stopping their growth and exposing them to pulling forces. Along the non-adhesive edges of the cell the microtubules continue to grow without binding to the cortex until they reach the next adhesive zone [89].

The natural polarization of cells depends on the ability of protein complexes to separate at different cell edges, with PAR proteins playing the most important role, while the rest of the proteins migrate through the cell, integrating into one or the other complex [90,91]. Dynein is needed for polarization and directional movement of fibroblasts [92]. The shift of dynein to the front of the cell is regulated by FAK kinase [93] and with the participation of cdc42, polarity protein Dlg1, which interacts with dynein via the scaffolding protein GKAP [94]. In addition, some authors have paid attention to local changes in the dynamics of microtubules, which can occur, for example, at the leading edge of fibroblasts moving along the substrate [95,96,97]. Such changes can influence pushing forces elaborated by microtubules. In particular, the paxillin focal contact protein inhibits HDAC6 deacetylase and increases microtubule acetylation in the front of the cell, which leads to the shifting of the centrosome and Golgi apparatus there [98]. The actomyosin system also plays a role in the movement of the centrosome. With the participation of DISC1 (disrupted in schizophrenia 1), a phosphorylated form of myosin II accumulates in the centrosome. This is necessary for proper centrosome orientation and radial migration of developing mouse neurons [99]. Such a myosin can interact with centrosomal actin.

In the epithelial sheets, the centrosome is held in the apical part of the cell by microtubules associated with intercellular adhesions and, possibly, by the apically located actin cortex. Obviously, for the apical displacement of the centrosome, it is first necessary to establish the basal-apical polarization of the cell. For the formation of the lumen of the ducts (at least in the culture of renal cells), certain ECM properties and tight cell packing are necessary [100]. During lumen formation, GTPase cdc42 accumulates on the apical membrane. Interestingly, the activity of cdc42 in the plasmalemma is necessary for the proper organization of the centrosome, including centrioles and pericentriolar material [101]. In the apical membrane, cdc42 attracts a protein complex that includes Crumbs (CRB), Stardust (Pals1), PatJ, PAR-6, ERM (ezrin, radixin, moesin), merlin, atypical protein kinase C (aPKC), and some other proteins [82,102]. The apical complex displaces PAR-3 (partitional defecting protein 3)/Bazooka (Baz) protein to lateral surfaces of cells where it is involved in the formation of adherens junctions in conjunction with the specific components of these junctions. Microtubules extending from the centrosome are fixed at adhesions, especially astral microtubules in mitosis [43], but microtubules extending from apically displaced interphase centrosome can be fixed as well. In fact, the detailed mechanism of apical displacement of the centrosome has not been studied; it was shown that actin-binding proteins ERM and merlin are involved in displacement [80]. Interestingly, ERM and merlin similarly provide centrosome clustering in the case of the presence of multiple centrosomes in the cell, which confirms that these proteins participate in the centrosome movement through the cell [80], although the mechanism of this participation is obscure. Actin-dependent apical centrosomal displacement is necessary for their subsequent proximal-distal polarization during PCP development [54].

The rearrangements of the nucleus-centrosome axis during epithelial-mesenchymal transition (EMT) accompany carcinogenesis and a number of events during embryogenesis, for example, the development of the ducts of the mammary gland or renal ducts, where individual cells migrate from the epithelial layer to the surrounding ECM. As early as 1989, it was described that during EMT, the centrosome moves together with the Golgi apparatus to the basal part of the cell, and intercellular adhesions are disassembled; then, filopodia penetrating the basal lamina are formed and the cell acquires mesenchymal (fibroblast-like) features [103]. The basal part of the cell becomes anterior during further migration through the ECM [23]. A phenomenon similar to EMT was reproduced in vitro by placing cells in microfabricated substrate patterns in pairs. In patterns, contacts between cells form; there were also contacts of cells with a substrate along the periphery. It turned out that in paired MCF-10A cells (mammary gland cells), MDCK (kidney cells), and several other types of epithelial cell centrosomes are usually located near cell contact; however, under the influence of known EMT inducers TGF-β or HGF centrosomes move outward, and the distance between them increases sharply (Figure 1F–H). This cell’ reaction simulates EMT; it is suppressed by a TGF-β receptor inhibitor. If you create an adhesive substrate around the cell, then under the action of TGF-β or HGF cells “scatter” from each other. Polarization and scattering are accompanied by a major restructuring of the microtubule system, in which the statin protein sequestering tubulin dimers is activated: microtubules become smaller and more dynamic. As we remember, this helps move the centrosome and its aster through the cell. Artificial stabilization of microtubules prevents the polarization and scattering of cells. Par3 also participates in EMT; in paired cells, it is localized in an intercellular junction, and excess of Par3 prevents the centrosome from being repositioned by TGF-β, though its depletion leads to centrosome scattering [23].

Displacements of the centrosome during the formation of the primary cilium or immune synapse are accompanied by the accumulation of dynein in the region of the plasmalemma where the centrosome is displaced. The accumulation of dynein is due to diacylglycerol in the plasmalemma and, reciprocally, to the distribution of myosin [104]. The protein complex at the end of the microtubule (+TIP) is also activated and contributes to the capture of microtubules by dynein by the search and capture mechanism [105] and the development of pulling forces [106,107]. This activation involves activity of casein kinase delta, phosphorylating EB1 protein, itself activated through signal transduction from receptors [108]. Interestingly, during the formation of the immune synapse, the stability of microtubules does not play a significant role in the movement of the centrosome; more important is the change in the arrangement of microtubules [109]. Vesicular transport proteins play a significant role in the formation of the immune synapse and the primary cilia [38]. It can be assumed that the centrosome shift depends on the dynein activated on the vesicles, but the experimental work on this topic remained unknown to us, despite our search efforts. A significant role in the formation of the immune synapse is also played by the rearrangement of actin filaments. Upon lymphocyte activation, the Arp2/3 complex moves from the centrosome to the immune synapse, which leads to the detachment of the centrosome from the nucleus and facilitates its movement to the synapse region [110].

An analysis of the molecular mechanisms of centrosome displacement from a centroid during PCP requires an integral assessment of the asymmetry in the centrosome arrangement. Garrido-Jimenez et al. [54] proposed ‘representative polarized centriole distribution’ (RPCD), which estimates the position of a centrosome in two dimensions in a plane orthogonal to the basoapical axis. The assessment is based on the analysis of a large number of cells, which allows us to calculate the region of the most probable location of the centrosome in a particular experiment and then compare the experimental results. It has now been established that PCP depends on two signaling pathways, one of which is activated at the proximal edge of the cell and the other at the distal edge of the cell, and its onset is associated with intercellular contacts. This signaling is performed by the Frizzled/Dishevelled/Flamingo/Diego and the Vang/Prickle/Flamingo complexes, with their *Drosophila* Planar Polarity Effector (PPE) and vertebrate Ciliogenesis and PLANar polarity Effector (CPLANE) [111]. The components of the signaling pathways are delivered to the cell edges via microtubules [112,113], which suggests a connection with the centrosome position [24]. Both signaling pathways affect the dynamics of actin and its location in cells and are mainly studied in relation to the polarized arrangement of hairs on the *Drosophila* wing cells [111]. In turn, the centrosome position also depends on this signaling [30,54], probably, with a positive feedback. Interestingly, CPLANE is involved in ciliogenesis, which shows the relationship between ciliogenesis, the dynamics of actin filaments, and the location of centrosomes. In *Drosophila*, adherens junctions are symmetrically located in the cells during gastrulation, but this symmetry can be lost, and PCP can occur when aPKC is inhibited and the connection of microtubules to centrosome is strengthened [114]. This may mean that the centrosome itself organizes its shift at PCP by positive feedback through microtubules-adherens junctions-pulling force. Actin filaments are involved in the centrosome movement to the apical region of the cell [115]; however, they do not participate in centrosome displacement within the framework of PCP [54]. The mechanism of centrosome polarization in PCP remains unclear.

One more example of centrosome shifting in cells is determination of the spindle pole positioning in stem cells mitoses. This phenomenon was discovered in pioneering works of Yukiko Yamashita [116,117]. It consists in the fact that a stem cell, for example, *Drosophila* male germline cell, closely adheres to its niche (epithelium), and divides strictly perpendicular to it. In this case, the proximal daughter cell remaining in the niche remains the stem, and the distal cell begins to differentiate. In such cells, there is a checkpoint of centrosome orientation, and they do not begin division until the spindle poles take their proper (proximal and distal) position [118]. Interestingly, cells distinguish between centrosomes containing the maternal or daughter centrioles of past mitosis, placing them proximally and distally, respectively. Centrosome positioning in this system occurs with the participation of external signals emanating from niche cells: cytokine receptor homolog Dome, acting downstream of the niche-derived ligand Upd, directly binds to the microtubule-binding protein Eb1 to regulate spindle orientation [117]. Internal cellular mechanisms, including asymmetric distribution of Par-3/Bazooka protein and the APC cancer suppressor in the cell cortex, also participate in spindle orientation. Their interaction with maternal or daughter centrioles requires further study.

Last but not least, a centrosome commits multiple displacements at spermatogenesis. It has been shown that in insect spermatids, the centrosome first binds to the nucleus, and then, the flagellum grows toward the cell membrane [119]. Conversely, in vertebrate spermatids, the centrosome first migrates to the cell surface, where it forms a flagellum, and only later the nucleus approaches the centrosome [120].

## 4. Conclusions

The location of the centrosomes in the cell should be estimated in volume, along three orthogonal axes. In the absence of external cues, the centrosome tends to occupy a centroid of the cell if the nucleus does not interfere with it. The centroid is determined minus the periphery actin cortex. Centrosome central position is facilitated by the pulling (from the side of the dynein) and pushing (from the side of the dynamics of microtubules) forces applied to the microtubules, as well as the forces developed by actomyosin, which are not yet fully understood. The centrosome can be shifted from the center when signaling pathways are turned on; a shift of the centrosome is facilitated by changes in the dynamics of microtubules and their capture at the edges of cells, the redistribution of dynein, probably including the redistribution of vesicular structures on which dynein can be accepted, and the redistribution of actomyosin.

## Figures and Tables

**Table 1:** Position of centrosome in culture cells with local microtubule disruption (LMD) (data from [58]).

Experimental Conditions	Dynein Status	Myosin Status	Microtubule Dynamics	LMD	Centrosome Position
**Control**	active	active	intact	-	Centering
**Myosin inhibition**	active	inhibited	intact	-	Centering
**Dynein inhibition**	inhibited	no matter	intact	-	Severe decentering
**Dynein inhibition, microtubule stabilization**	inhibited	no matter	microtubules stabilized	-	Centering
**Local nocodazole application**	active	active	intact in the opposite side of the cell	+	Shift to LMD region
**Local nocodazole application, myosin inhibition**	active	inhibited	intact in the opposite side of the cell	+	Shift away from LMD region
**Dynein and myosin inhibition**	inhibited	inhibited	intact	-	Oscillations around cell center
